# Comparison of Safety and Outcomes between Endoscopic and Surgical Resections of Small (≤ 5 cm) Primary Gastric Gastrointestinal Stromal Tumors

**DOI:** 10.7150/jca.29443

**Published:** 2019-07-10

**Authors:** Taohong Pang, Yan Zhao, Ting Fan, Qingqing Hu, Dekusaah Raymond, Shouli Cao, Weijie Zhang, Yi Wang, Bin Zhang, Ying Lv, Xiaoqi Zhang, Tingsheng Ling, Yuzheng Zhuge, Lei Wang, Xiaoping Zou, Qin Huang, Guifang Xu

**Affiliations:** 1 Department of Gastroenterology, Affiliated Drum Tower Hospital of Nanjing University Medical School.; 2 Department of Gastroenterology, Affiliated Chaohu Hospital of Anhui Medical University.; 3 Department of Geriatric, Affiliated Drum Tower Hospital of Nanjing University Medical School.; 4 Department of Gastroenterology, Nanjing Drum Tower Clinical College of Nanjing Medical University.; 5 Department of General Surgery, Affiliated Drum Tower Hospital of Nanjing University Medical School.; 6 VA Boston Healthcare System and Harvard Medical School, West Roxbury, MA 02132, USA.

**Keywords:** Gastric gastrointestinal stromal tumors, Endoscopic resection, Surgical resection, Operative complications, Postoperative course, Adjuvant therapy with imatinib

## Abstract

Background and aims: Endoscopic resection is increasingly performed for gastric gastrointestinal stromal tumors (GIST). However, the safety and outcomes remain elusive. We aimed in this retrospective study to compare operative complications and prognosis between endoscopically and surgically resected small (≤ 5 cm) GIST tumor groups.

Methods: In this single-center retrospective study, we compared demographics, clinical outcomes, and the R0 resection rate between the endoscopy (n =268) and surgery (n =141) groups. Only GIST tumors in size of ≤ 5.0 cm were recruited for this comparison study.

Results: Overall, the mean age of patients was 59.0 years (range: 31.0-83.0). The male-female ratio was 0.68. The most common site of GIST was, in the descending order, the gastric fundus (55%), corpus (27.6%), cardia (10.8%), and antrum (6.6%). Compared with the surgery group, GIST tumors in the endoscopy group were significantly smaller (1.69±0.9 cm, *vs*. 3.20±1.2 cm in the surgery group; P <0.001) in size; postoperative hospital stay was significantly shorter (4.66±1.5 days, *vs.* 8.11±5.0; P <0.001); post-resection time to first liquid diet was significantly shorter (1.94±1.1 days, *vs.* 4.63±2.6; P < 0.001); the incidence of operative and post-operative complications was significantly fewer (p < 0.05), and hospital costs were significantly lower (20115.4±5113.5¥, *vs.* 43378.4±16795.7¥; P < 0.001). The R0 resection rate was significantly lower in the endoscopy (93.3%) than in the surgery (99.3%) groups (P< 0.01). In the endoscopy group, 176 (65.7%) and 69 (25.7%) patients were found to be at very low and low risk of aggressiveness, respectively, in comparison to 27(19.2%) and 86 (61.0%) patients in the surgery group, respectively (P <0.001). Among 409 cases, 50 (12.2%) were found to be at intermediate or high risk of aggressiveness, 20 of which were treated with adjuvant imatinib therapy and but only 8/20 taking imatinib for 1 to 3 months because of side effects and high costs. No local or distant tumor recurrence was observed over an average of 33.5-month follow-ups. Two patients died of other disease in the surgery group.

Conclusions: Endoscopic resection of selected small gastric GISTs (≤ 5cm) was feasible, safe, and associated with better intraoperative results and an equal postoperative course, compared to surgical resection.

## Introduction

Gastrointestinal stromal tumors (GISTs) are the most common mesenchymal tumors in the gastrointestinal (GI) tract 1. In general, GISTs tend to occur more frequently in older male or female patients 2, and the stomach is the most common site with more than half of all GISTs 3, 4. Pathogenetically, more than 90% of GISTs are due to mutations in the KIT or platelet-derived growth factor receptor α gene 5-7. The traditional therapy for GISTs is open surgical or laparoscopic resection 8-13. However, surgical therapy has inherited some unavoidable disadvantages, such as considerable iatrogenic disruption of the normal GI tract integrity, longer operative time, more blood loss, slower functional recovery, a relatively longer postoperative hospital stay, and higher hospital cost 14,15. In this regard, endoscopic therapy appears to have several advantages over surgical therapy because of minimal injury and negligible disturbances in the GI tract function, which is especially attractive for elderly patients and the patients who could not tolerate surgical resection because of poor health. In fact, more than 50% of patients with surgically resected GISTs were found to have very low and low risk for complications 16, 17. These patients would be excellent candidates for endoscopic, rather than surgical, resection of their GIST tumors. However, a comparison study on operative complications and post-resection outcomes between endoscopic and surgical resections of GIST remains scarce.

Endoscopic resection includes endoscopic mucosal resection (EMR) and endoscopic submucosal dissection (ESD) and has become a standard treatment modality for intestinal-type intramucosal early gastric carcinomas 18. Most recently, more advanced novel endoscopic resection techniques, such as endoscopic submucosal excavation (ESE), submucosal tunnel endoscopic resection (STER), and endoscopic full-thickness resection (EFTR) have been developed and successfully applied to *en bloc* endoscopic resection with curative intend for submucosal tumors in the upper GI tract 19-25. Zhou *et al*. 22 reported to use the EFTR technique for a complete (100%) endoscopic resection of 26 gastric GISTs without laparoscopic assistance. In that study, the average size of GISTs was 2.8 cm (range, 1.2-4.5). There were no major operative and post-resection complications such as bleeding, peritonitis, or abdominal abscess at up to 8 months of follow-up. Similarly, Wang et al. 25 employed the STER technique to successfully resected 57 submucosal tumors in the gastroesophageal junction region with the mean tumor size of 2.15 cm (range: 0.6-3.5). The *en bloc* resection rate was 100 %. No delayed bleeding or severe adverse events were reported in any patients. No local recurrence and distant metastasis occurred during a 24-month follow-up period. However, despite satisfactory endoscopic resection of gastric GISTs with conventional ESD or novel EFTR/STER techniques, there have been no systematic comparison studies on the safety and post-resection clinical outcomes between endoscopic and surgical resections of small (≤5.0 cm) gastric GIST tumors.

The aim of this study was to evaluate the safety and outcomes of endoscopic resection of gastric GISTs, in comparison with those of surgical resection in 409 gastric GISTs resected at a single high-volume center in China.

## Materials and Methods

### Patient Selection and Groups

**Figure 1:**
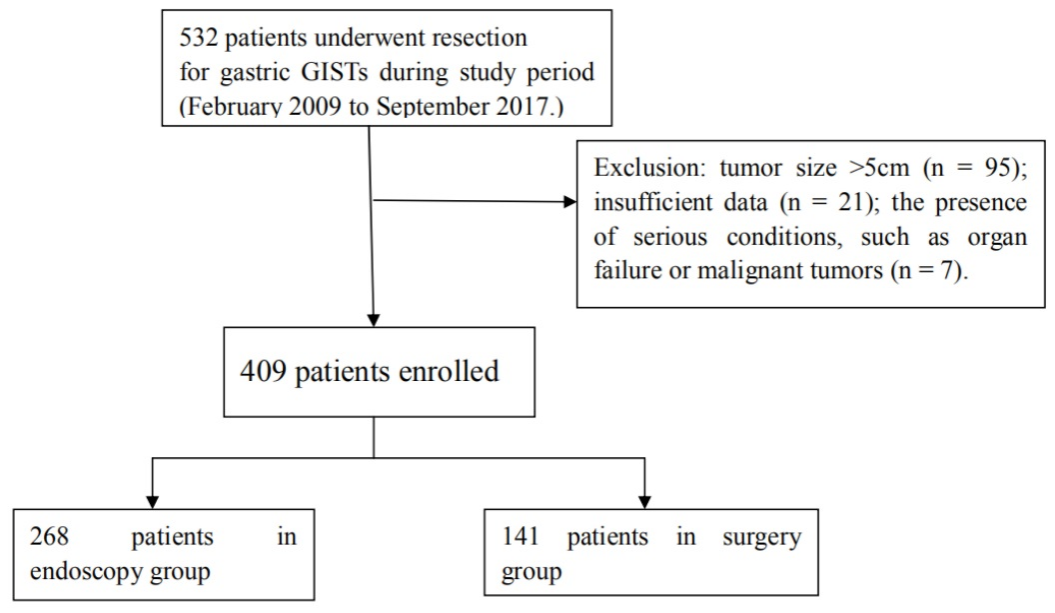
Flowchart for patient selection and grouping

A total of 532 patients with gastric GIST resections were identified in the medical records stored at the Nanjing Drum Tower Hospital affiliated to Nanjing University Medical School over the period from February 2009 to September 2017. The medical records of all resected GIST cases were reviewed and 123 were excluded because of the presence of sarcomatous malignancy or multi-organ failure (N=7), large tumor size of greater than 5 cm (N=95), or insufficient data (N=21). As a result, 409 cases with the tumor size of < 5 cm were included in the study, among which 268 tumors were resected endoscopically (the endoscopic group) and 141 tumors removed surgically (the surgical group) (Figure 1). Before resection of gastric GISTs, all patients underwent esophagogastroduodenoscopy (EGD) and endoscopic ultrasonography (EUS) to estimate the tumor size, location, endoscopic appearance, the origin in the gastric wall, and the presence or absence of other lesions. A routine computed tomography (CT) was utilized to evaluate the presence or absence of metastatic diseases and the tumor growth pattern. This study protocol was approved by the Medical Ethics Committee of the Nanjing Drum Tower Hospital.

### Endoscopic Resection

**Figure 2:**
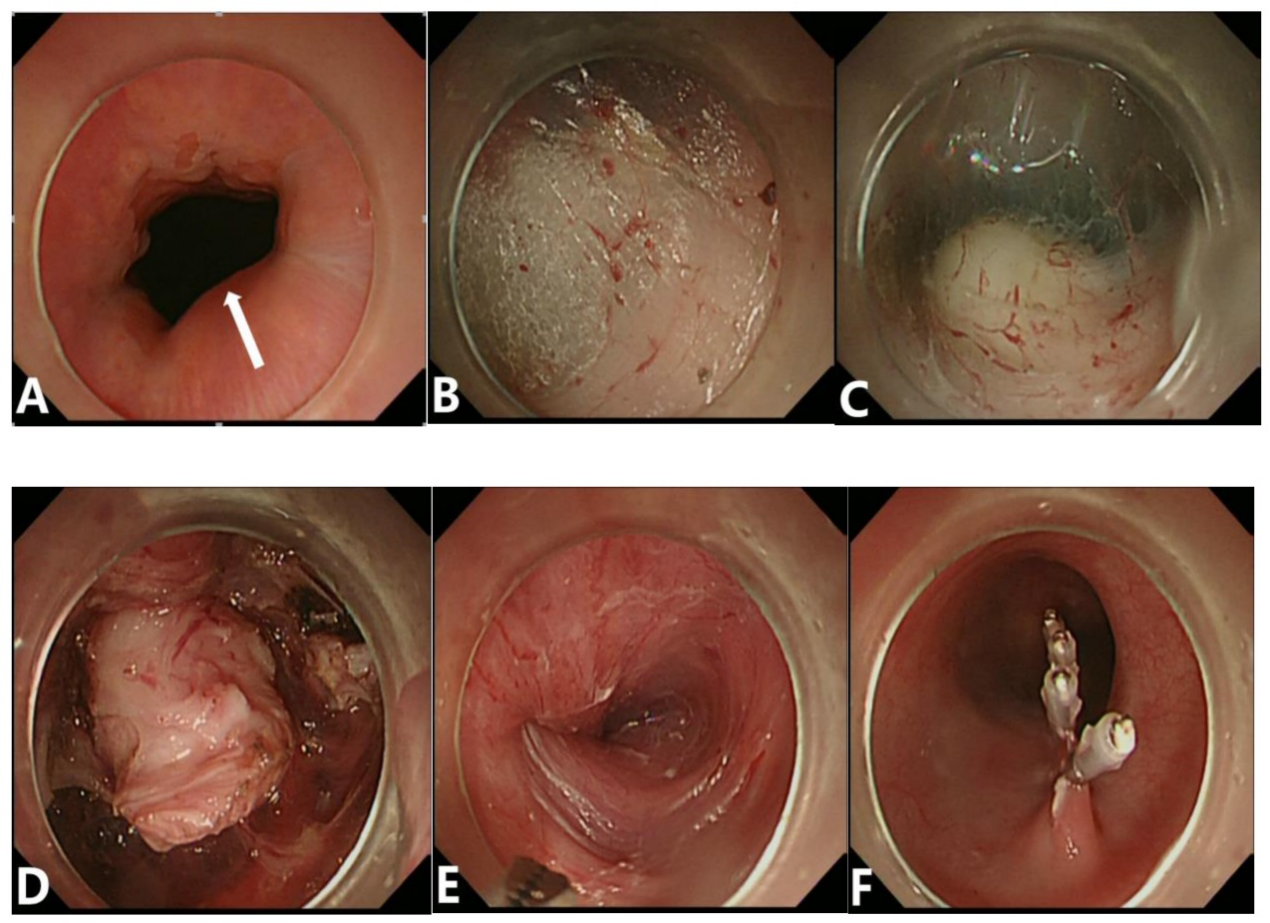
Procedures of STER. (A) Endoscopic view of tumors located in the gastric cardia with a broad-based protruding growth pattern and a smooth surface in size of 1.3 x 1.0 cm. (B) Establishment of a submucosal tunnel. (C、D) A SET was exposed and completely resected. (E、F) After removal of the tumor, no major adverse events occurred and the wound was closed with clips.

**Figure 3:**
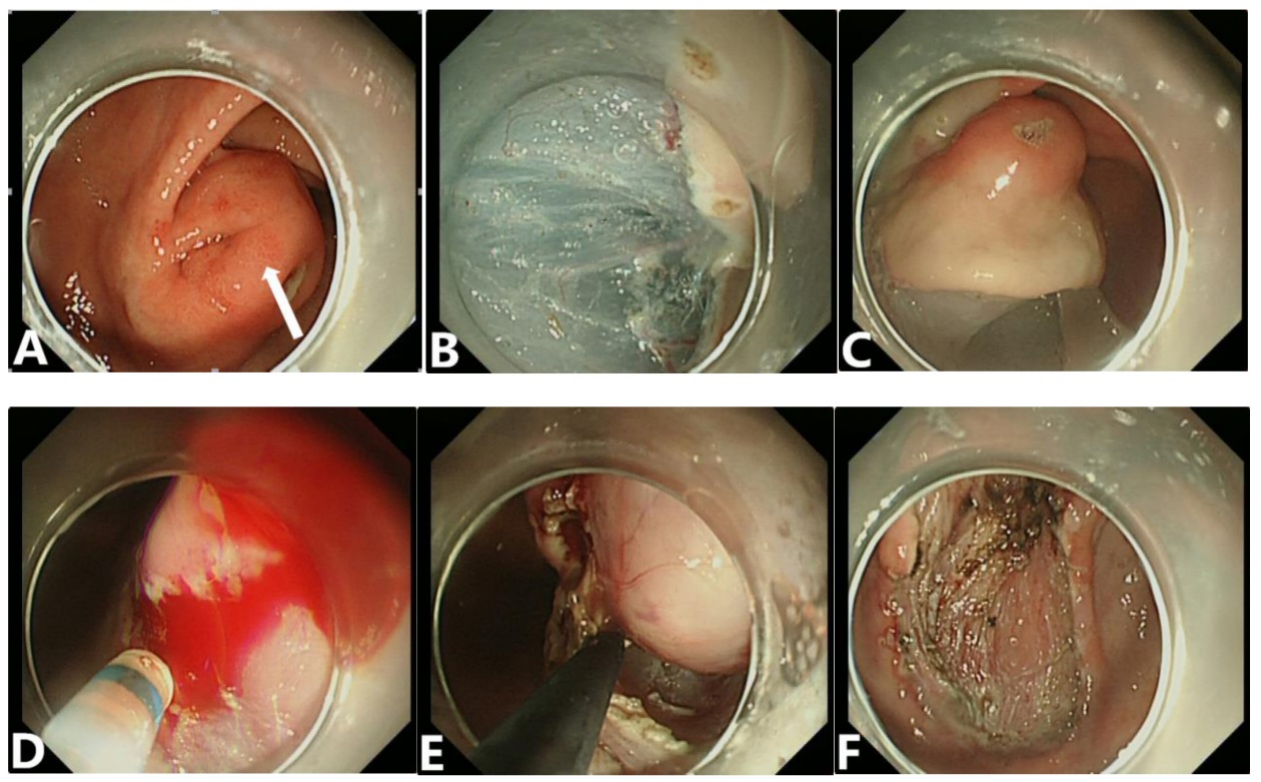
A gastric submucosal tumor (SET) resected by a standard endoscopic submucosal dissection method. (A) Endoscopic view of a tumor located in the gastric antrum with a broad-based protruding growth pattern. The tumor was large, measured 3.5 x 2.5 cm in size, and showed an uneven, non-bleeding, eroded mucosal surface (white arrow), and a mucous bridge. (B、C) After marking the circumferential border of this tumor and submucosal saline injection, a pre-cut incision was made. (D、E) The tumor was lifted and completely resected. (F) After removal of the tumor, no residual tumor nor bleeding lesions were identified at the tumor bed.

All patients were placed under general anesthesia for resection of their GIST tumors and divided into either the endoscopic group or the surgical group. In the endoscopic group, all endoscopic resections were performed by skilled gastrointestinal endoscopists with a standard endoscopic therapy protocol (Figures 2,3). In brief, the patient was sedated with propofol (1.0 mg/kg) or midazolam (0.035 mg/kg) and the cardiorespiratory functions were continuously monitored throughout the entire procedure. After injection of 0.9% saline solution, containing epinephrine (1:10,000) and the indigo carmine dye, into the submucosal layer, the endoscopist completely separated the GIST tumor from the surrounding gastric tissue with a Dual knife (KD-650L; Olympus Optical Co. Ltd) or insulated-tip knife (KD-610L; Olympus Optical Co. Ltd). Clips were used to close the incision to prevent bleeding and perforation. A complete endoscopic resection of gastric GISTs was defined as the absence of any residual tumor visible endoscopically after tumor resection.

The most common methods for 268 endoscopically resected GISTs were, in the descending order, endoscopic submucosal enucleation with ESD (N=136, 50.7%), EFTR (N=88, 32.8%), ESE (N=29, 10.8%), STER (N=12, 4.5%), and EMR (N=3, 1.2%).

### Surgical Resection

In the surgical group, all 141 patients received general anesthesia with tracheal intubation for either open surgical or laparoscopic resection of their GIST tumors. Laparoscopic or open resection was performed according to a standard procedure. Briefly, in the laparoscopic approach, the first trocar was placed in the midline near the umbilicus, 2 other ports were inserted in the right and left flanks, and the forth trocar was used to facilitate correct exposure of the surgical fields. For patients in the open surgical resection group, the procedure was started with a traditional midline upper abdominal incision. Subtotal or total gastrectomy was performed with a routine protocol. After opening of the abdominal cavity, the surgeons routinely inspected abdominal organs and peritoneum for possible GIST metastasis and lymphadenopathy. Frozen section diagnoses of the presence or absence of GIST tumor malignancy and the resection margin status were routinely carried out during the surgical resection procedure. On the basis of the GIST tumor size and location, the tumor was completely resected with laparoscopic local resection (N=67, 47.5%), open local surgical resection (N=44, 31.2%), open surgical wedge resection (N=9, 6.4%), subtotal gastrectomy (N=20, 14.2%), or in a rare case, total gastrectomy (N=1, 0.7%). One patient had total gastrectomy and lymph node dissection due to the preoperative CT impression of abdominal lymph node enlargement, suspicious for metastasis, which was not confirmed by postoperative pathology evaluation of resected specimens. In our study, among 20 patients underwent subtotal gastrectomies, 8 had concurrent gastric intraepithelial neoplasia, 5 had another submucosal tumor lesion discovered at the operative procedure, and 7 had tumors at difficult regions, such as cardia and pylorus. Many studies showed different surgical techniques are required depending on the tumor location and configuration to ensure a complete resection with a negative resection margin 26-29.

### Pathologic Examination

All resected GIST tumors were immediately fixed in 10 % buffered formalin solution with a standard surgical pathology specimen processing protocol for pathological evaluation. The mitotic index (MI) was defined as the number of mitosis per 50 high-power fields (HPF) on a routine hematoxylin-eosin stained tumor section. The risk potential of GISTs was categorized by the largest tumor dimension and the mitotic index (Table 1), according to a consensus protocol 30. A pathologic diagnosis of GIST was confirmed with characteristic histopathologic features and immunohistochemical staining patterns of neoplastic cells to CD117 (c-kit), CD34, DOG1, smooth muscle actin (SMA), S-100, and Ki-67. All histopathologic assessments were performed by 2 gastrointestinal pathologists with minimal disagreements which were resolved after a joint review of slides to reach a consensus.

### Resection-associated Complications and Margins

During (early) and after (late) GIST resections, procedure-related complications were recorded and compared between the endoscopic and surgical groups. The complications included: 1) bleeding, defined as > 200 ml of fresh blood loss, 2) acute infection, defined as post-operative fever with the body temperature over 38°C and the presence of bacteria growth in blood or secretion culture, 3) perforation, defined as direct endoscopic observation of mesenteric tissue beyond the GI tract, and 4) pneumoperitoneum, defined as detection of free air by a simple abdominal radiograph or CT scan.

The status of the resection margin was assessed pathologically and routinely categorized as R0, referring to the absence of residual GIST tumor cells at the inked resection margins; R1, defined as the microscopic presence of tumor cells at the inked resection margins; and R2, defined as the gross presence of a residual tumor at the resection margins.

### Post-resection Outcome Evaluation

Under a conventional endoscopic therapy protocol, all patients were routinely subjected to a post-EGD follow-up endoscopic examination 3 months after the GIST resection. A surveillance upper endoscopy procedure was performed annually thereafter for 3 years. In some cases, a CT scan was utilized to evaluate any recurrence or metastasis of GIST tumors. For patients who underwent surgical resection, surveillance upper endoscopy along with a routine CT scan were carried out semi-annually for the first year, and then annually for 3 years.

### Statistical Analysis

All data management and statistical analyses were carried out with the SPSS statistical program (SPSS 22.0, Chicago, IL, USA). Continuous data were expressed as mean ± standard deviation (SD). Associations involving continuous data were assessed with the two-tailed Student *t* test. Discontinuous data were analyzed with the Chi-square or Fisher's exact test. Baseline clinicopathologic characteristics were not balanced between the endoscopy and surgery groups. Thus, we applied a propensity score matching analysis, which is used to minimize potential selection bias and mimic randomization in observational studies 31. The cumulative survival rate was estimated by the Kaplan-Meier analysis with a log rank test. A *p* value <0.05 was considered statistically significant.

## Results

### 1. Patient Demographics and Tumor Size and Location

As shown in Table 2, there was no significant difference in patient age between endoscopic and surgical groups. A significantly higher percentage of GIST tumors was found in female patients than in male patients (*p* < 0.05). Thus, the overall male-female ratio was 0.68. The average tumor size was 2.23 cm (range 0.4-5.0cm). Compared to the surgical group, the tumor size was significantly smaller in the endoscopic group (*p* < 0.0001). The overall distribution of tumor location was significantly different with the most common site, in a descending order, in the fundus, corpus, cardia, and antrum between endoscopic and surgical groups.

### 2. Early Resection Complications

As shown in Table 3, early resection-associated complications were significantly lower in the endoscopy group (N=9, 3.3 %) than in the surgery group (N=15, 10.7 %) (*p*<0.05). Intra-operative bleeding was absent in the endoscopy group but present in 8 (5.7%) cases in the surgery group. However, postoperative hemorrhage (N=3, 1.1%), perforation (N=1, 0.4%), and pneumoperitoneum (N=3, 1.1%) occurred only in the endoscopy group, but not in the surgery group, while acute infection was discovered in only 2 (0.7%) cases in the endoscopy group, compared to 7 (5%) cases in the surgery group. All complications were successfully managed conservatively, except for one late perforation case that was repaired surgically.

Overall, the resection margin involvement of residual GIST tumors was significantly more common in the endoscopic group than in the surgical group, as shown in Table 3, with R0 and R1 resections of 93.3% and 6.7%, respectively, for the endoscopy group, compared to 99.3% and 0.7%, respectively, for the surgery group.

**Figure 4:**
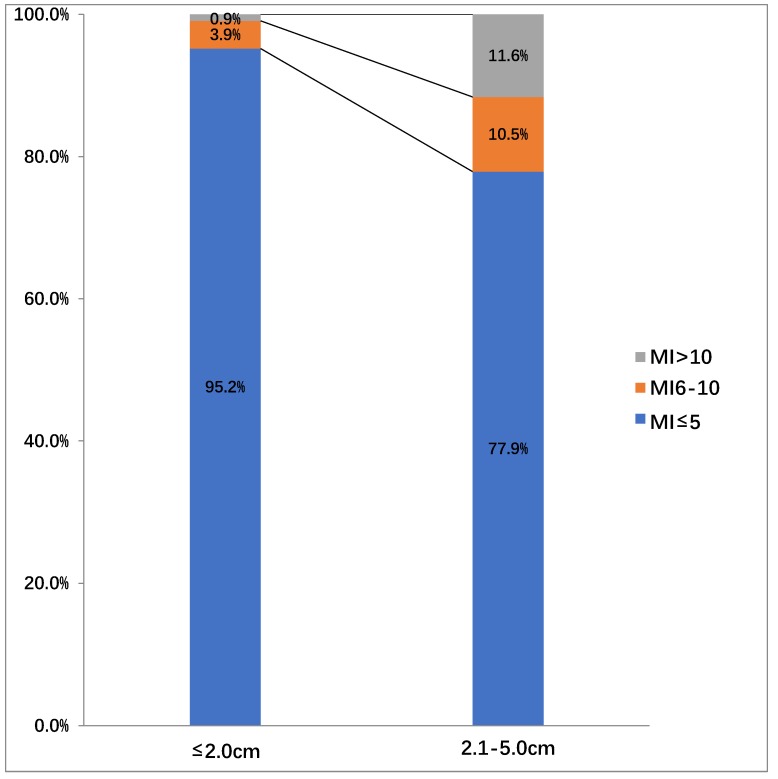
The distribution of the mitotic index (MI) among all 409 patients according to tumor sizes.

In the endoscopy group, 176 (65.7%), 69 (25.7%), 14(5.2%), and 9 (3.4%) patients were found to be in very low, low, intermediate, and high risk of aggressiveness groups, respectively. In contrast, 27(19.1%), 87(61.7%), 14(10.0%), 13(9.2%) patients were found to be in very low, low, intermediate, and high risk of aggressiveness groups in the surgery group, respectively. The risk stratification was significantly different between the endoscopy and surgery groups (*p* <0.001). We also assessed the difference in the mitotic index among all 409 patients, according to tumor sizes (Figure 4). In patients with a tumor size ≤ 2.0 cm, the mitotic index was ≤ 5 per 50 high-power fields (HPF) (217/228, 95.2%) in the vast majority of cases, and the number of those with the mitotic index between 6-10 (3.9%, 9/228) and >10 (0.9%, 2/228) per 50 HPF were very small. In contrast, In patients with tumor sizes between 2.1 cm and 5.0 cm, the number of patients with the mitotic index between 6-10/50 HPF (10.5%, 19/181) and >10/50 HPF (11.6%, 21/181) was increased. However, the number of patients with the mitotic index ≤ 5/50 HPF was the largest (77.9%, 141/181) among the three types. Nine patients (3.9%, 9/228) were in the intermediate risk group and 2 (0.9%, 2/228) patients were in the high risk group, in which the tumor size in both cases was ≤2.0 cm.

Among 50 patients, only 20 had adjuvant therapy with imatinib after resection. Imatinib was administered to 10 patients in each group, in which 8 (5 in the endoscopy group and 3 in the surgery group) took imatinib only for 1 to 3 months because of severe side effects and high costs. In the other 12 patients, imatinib treatment was carried out for 1 or 3 years in 5 patients in the endoscopy group (3 for 1 year and 2 for 3 years) and 7 in the surgery group (5 for 1 year and 2 for 3). The difference was not significant between the two groups (Table 4).

### 3. Post-resection Hospital Course

As shown in Table 5, the gastric functional recovery time was significantly shorter in the endoscopy group than in the surgery group (*p* < 0.0001). Thus, the post-resection hospital stay (*p* < 0.0001) was significantly shorter and the overall hospital cost (*p* < 0.0001) was significantly lower in the endoscopy group than in the surgery group.

### 4. Comparisons of the propensity score matching analysis

As shown in Table 6, there were no significant differences in demographics and tumor size between the endoscopy and surgery groups. After the tumor size was matched between the two groups, the results in the endoscopy group were superior to those in the surgery group. Overall, the endoscopy therapy showed significant advantages over the surgery therapy in shorter postoperative hospital stay (p < 0.001) and the post-resection time to the first liquid diet (p < 0.001), fewer incidences of operative and post-operative complications (p=0.026), and lower hospital costs (p<0.001).

### 5. Late Complications and Prognosis

**Figure 5:**
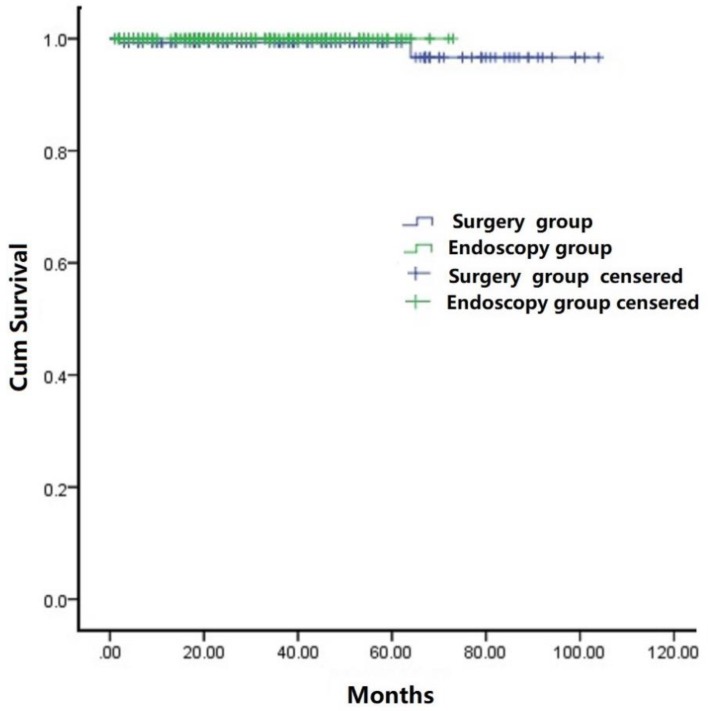
Kaplan-Meier analysis of overall survival of patients treated with either endoscopy therapy or surgery therapy.

Overall, the mean follow-up period was 33.5 months (range: 3 - 104) for the cohort. During the follow-up period, no local or distant tumor recurrence was observed in any patients. Two patients died of other GIST-resection unrelated diseases in the surgery group. The cumulative survival rate (Figure 5), as estimated by the Kaplan-Meier analysis, was not statistically significantly different between the endoscopic and surgical groups (*p* =0.509).

## Discussion

GISTs, although rare, are the most common mesenchymal tumor of the digestive tract 32, 33. These tumors occur primarily in the stomach, but also in small intestine, and even in extra-intestinal locations including the omentum and peritoneum 32-35. Currently, the practice guidelines of the United States National Comprehensive Cancer Network recommend all GIST tumors in size of > 2.0 cm be resected, whereas for GISTs in size of < 2.0 cm, the guidelines are indecisive and suggest either resection or surveillance 28. Since every GIST tumor is potentially malignant if the tumor shows a high mitotic index 36, as demonstrated in our study with 9 such small tumors, all GISTs should be resected once discovered, regardless of tumor size, given the high cost for the long-term surveillance of patients with small GISTs 37. In this regard, the endoscopic approach for small GIST tumor resections would be an attractive alternative treatment option in selected cases. In our cohort of 181 patients with a tumor size of 2.1-5.0 cm, 141 had the mitotic index of ≤ 5, similar to that reported by Joo *et al*. 16 These small GIST tumors may be the ideal candidates for local excision by minimal invasive resection procedures, such as endoscopic resection because of considerable advantages over surgery resection, such as faster recovery, shorter hospital stay, and lower cost, especially for tumors in the cardia or esophagogastric junction, which is a challenge for laparoscopic resection. The results of our study provide substantial evidence supporting this recommendation. After marching demographics and tumor size between the endoscopy group and surgery group, the results of multiple comparison parameters in the endoscopy group were superior to those in the surgery group, including shorter postoperative hospital stay and post-resection time to first liquid diet, fewer incidences of complications, and lower hospital costs.

In contrast, the surgical approach was superior to endoscopic therapy in resection of larger (>5 cm) tumors to achieving negative resection margins. In this study, the R0 resection rate was 93.3% in the endoscopy group, which was significantly lower than that (99.3%) in the surgery group (*p* =0.006). However, there were no local or distant tumor recurrences in either group over the follow-up period. This finding is similar to that reported previously 16, 38. DeMatteo *et al*. 38 demonstrated that GIST tumors in size >10 cm tend to recur early, but other factors, such as microscopic positive resection margins (R1 resection), did not influence recurrence outcomes. Moreover, Kim reported a local recurrence rate of 5.8%, even with R0 resection in large tumors 15. Based on our current and previous study data 16,38, we suggest that if a GIST tumor is completely resected endoscopically with a lower stratified risk for aggressive clinical outcomes, additional surgical resection may not be needed, even in cases with R1 resection, although those patients should be closely followed up in a surveillance program.

Despite minimal injury to the gastrointestinal tract 39, 40, endoscopic resection of GISTs remains controversial because of major complications such as perforation. The tumor growth pattern is one of the reasons that may result in perforation. According to the relationship between GIST location in the gastric wall, Kim divides GIST tumors into four types: Type Ⅰ GIST refers to a tumor with a very narrow connection with the proper muscle layer (PM), protruding into the gastric lumen, like polyps; Type Ⅱ tumor has a wider connection with the PM and protrudes into the lumen at an obtuse angle; Type Ⅲ lesion is located in the middle of the gastric wall; and Type Ⅳ tumor protrudes mainly into the serosal side of the gastric wall 15. Wei et al. reported that the incidence of a gastric-wall defect (GWD) after endoscopy therapy for type-Ⅲ and -Ⅳ tumors was significantly higher than that for type-Ⅰand -Ⅱ tumors (94.7% vs. 15.3%) 41. The location of gastric GISTs is another factor that may cause perforation. Jeong ID *et al.* 42 reported that the incidence of perforation at the fundus was higher than that of other location. In our study, one patient had postoperative perforation that was repaired surgically. To avoid this serious complication, preoperative examinations of the tumor by using EUS and CT to select suitable cases for endoscopic resection become necessary.

Hemorrhage is another common complication of endoscopic treatment of gastric GISTs, including intra- and post-operative hemorrhage. Intra-operative bleeding may not affect the success of endoscopic treatment. Heavy intra-operative bleeding can affect a clear visual observation of the operative field. The incidence of tumor-related hemorrhage in endoscopic therapy ranges from 0 to 38.7% 22,25,43-44. In this study, no heavy intra-operative hemorrhage was observed in patients in the endoscopy group. The different incidence of bleeding in patients reported in previous studies may be related to the following factors: 1) Preoperative examination and evaluation of a patient with potential coagulopathy; 2) operative proficiency in endoscopic resection; 3) different definitions of bleeding; 4) case selection bias. Post-operative bleeding is defined as showing clinical bleeding signs such as hematemesis, black stool, etc. within 1 to 30 days after surgery, or a drop of blood hemoglobin levels of greater than 20g/l, or bleeding detected at follow-up endoscopic examination 45. Wei *et al*. 41 used ESD to resect 168 GISTs located in the muscularis propria layer, and reported delayed bleeding in 2 patients (1.2%), both of whom were successfully managed endoscopically. Joo *et al*. endoscopically resected 90 GISTs, and major bleeding was observed in only 1 patient, who was eventually managed surgically 16. In our study, 3 patients experienced postoperative hemorrhage, one of whom underwent endoscopic hemostasis with hemostasis clips, and the other two patients were treated conservatively without additional surgery.

Management of patients with risk of aggressive clinical outcomes remains a challenging task. In patients with high-risk GISTs, the postoperative administration of imatinib may decrease the recurrence and metastasis of GIST, as shown in our current and other previous studies 46-48. In patients with intermediate or high risk GISTs, our present study showed the absence of local or distant recurrence in our patients with or without postoperative imatinib therapy during the follow-up period. This result is inconsistent with the previous report. The discrepancy may be related to a short follow-up period and the small sample size in our cohort. A follow-up investigation for all such cases is on-going.

There are several limitations in our study. First, it was a single-center retrospective study with a non-randomized design. As such, selection bias might exist. However, we used a stringent study protocol with a consecutive patient selection process and a uniform exclusion method to minimize, if any, selection bias. The tumor size was balanced between the endoscopy group and surgery group by the propensity score matching analysis. Second, the duration of the follow-up period was relatively short, as mentioned above. Therefore, our study findings need to be verified with multi-center, prospective studies in large sample sizes to establish the role of endoscopic therapy in the treatment of small GIST tumors.

In conclusions, our study showed that endoscopic resection was safe and cost-effective for small (≤5 cm)) gastric GIST tumors with advantages of fewer complications, more rapid recovery, shorter hospital stay, and lower costs, compared to surgical resection, although perforation and bleeding did occur in few cases during and after endoscopic resection. Such complications were managed successfully without serious consequences. The overall clinical outcomes of endoscopic resection were favorable. The results need to be verified in larger studies.

## Figures and Tables

**Table 1:** Proposed modification of NIH consensus classification criteria for defining risk of aggressive clinical course of primary GISTs 30

Risk category	Tumor size(cm)	Mitotic index (per 50 HPFS)	Primary tumor site
Very low risk	<2.0	≤5	Any
Low risk	2.1-5.0	≤5	Any
Intermediate risk	2.1-5.0	>5	Gastric
High risk	<5.0	6-10	Any
	5.1-10.0	≤5	Gastric
	Any	Any	Tumor rupture
	>10	Any	Any
	Any	>10	Any
	>5.0	>5	Any
	2.1-5.0	>5	Non-gastric
	5.1-10.0	≤5	Non-gastric

**Table 2:** Comparison of Demographics, Tumor Size and Location between Endoscopic and Surgical Groups.

	Total(n=409)	Endoscopy group(n=268)	Surgery group(n=141)	P value
Age (year, mean±SD)	59.0±9.8	58.9±9.5	59.1±10.8	NS
Gender (number, %)				< 0.05
Male	166(40.6)	97(36.2)	69(48.9)	
Female	243(59.4)	171(63.8)	72(51.1)	
Male-Female Ratio	0.68	0.57	0.96	
Tumor size (cm) (mean±SD)	2.23±1.2	1.69±0.9	3.20±1.2	<0.0001
Tumor location (number, %)				<0.0001
Cardia	44(10.8)	35(13.1)	9(6.4)	
Fundus	225(55.0)	158(58.9)	67(47.5)	
Corpus	113(27.6)	64(23.9)	49(34.8)	
Antrum-pylorus	28(6.6)	11(4.1)	16(11.3)	

NOTE: SD: standard deviation; NS: not significant.

**Table 3:** Clinicopathological Outcomes of GIST Resection

	Total Number (%)	Endoscopy group (%)	Surgery group (%)	P value
Total number	409	268	141	
Complication	24(5.9)	9(3.4)	15(10.6)	<0.05
Intraoperative major bleeding(≥ 200ml)	8(2.0)	0(0)	8(5.7)	
Postoperative hemorrhage	3(0.7)	3(1.1)	0(0)	
Postoperative perforation	1(0.2)	1(0.4)	0(0)	
Infection	9(2.2)	2(0.7)	7(5.0)	
Pneumoperitoneum	3(0.7)	3(1.1)	0(0)	
Resection margin				<0.01
R0	390(95.4)	250(93.3)	140(99.3)	
R1	19(4.6)	18(6.7)	1(0.7)	
R2	0(0.0)	0(0.0)	0(0.0)	

**Table 4:** Comparison of Adjuvant Imatinib-treated Patients with Intermediate or High Risk of Aggressiveness between Endoscopy and Surgery Groups.

Treatment Duration	Total Number	Endoscopy Group	Surgery Group	P Value
				0.591
Total Number	50	23	27	
None	31(62.0)	13(56.5)	17(63.0)	
1 to 3 Months	8(16.0)	5(21.7)	3(11.1)	
1 Year	8(16.0)	3(13.0)	5(18.5)	
3 Years	4(8)	2(8.8)	2(7.4)	

**Table 5:** Comparisons of Post-resection Hospital Course

	Endoscopy group (mean±SD)	Surgery group (mean±SD)	P value
Total Number	268	141	
Time to first fluid diet(days),	1.9±1.1	4.6±2.6	<0.001
Postoperative length of hospital stay (days)	4.7±1.5	8.1±5.0	<0.001
Hospitalization expenses (RMB)	20115.4±5113.5	4.3378.4±16795.7	<0.001

Note: RMB: Renmingbi (The Chinese currency)

**Table 6:** Comparisons of results between Endoscopic and Surgical Groups after the propensity score matching analysis.

Resection Outcome	Endoscopy group	Surgery group	*P* value	
	(%)	(%)		
Total Number	84	84		
Age (year, mean±SD)	59.5±10.5	59.1±10.3	0.818	

Gender			0.276	

Male	33(39.3)	40(47.6)		
Female	51(60.7)	44(52.4)		
Tumor size (cm)(mean±SD)	2.48±1.03	2.50±0.96	0.854	
Complication	3(3.6)	11(13.1)	0.026	
intraoperative major bleeding(≥200ml)	0(0)	5(6.0)		
Postoperative perforation	1(1.2)	0(0)		
Infection	1(1.2)	6(7.1)		
Pneumoperitoneum	1(1.2)	0(0)		
Resection margin			0.014	
R0	76(90.5)	83(98.8)		
R1	8(9.5)	1(1.2)		
R2	0(0.0)	0(0.0)		
Time to first liquid diet (days),	2.1±1.3	4.6±3.0	<0.001	
Postoperative length of hospital stay (days)	4.9±1.9	8.0±5.4	<0.001	
Hospitalization expenses (RMB)	21884.3±5960.2	40267.2±13954.2	<0.001	
